# Effectiveness of vestibular rehabilitation on postural balance in Parkinson’s disease: a systematic review and meta-analysis of randomized controlled trials

**DOI:** 10.1186/s12883-024-03649-5

**Published:** 2024-05-14

**Authors:** Carla Marineli Saraiva do Amaral, Samuel Brito de Almeida, Renata Parente de Almeida, Simony Lira do Nascimento, Rodrigo Mariano Ribeiro, Pedro Braga-Neto

**Affiliations:** 1 Division of Neurology, Department of Clinical Medicine, Faculty of Medicine, Federal University of Ceará, Rodolfo Teófilo – Fortaleza – Ceará, R.Prof. Costa Mendes Street – 4th floor, Fortaleza, 1608 Brazil; 2 Department of Health Sciences, Faculty of Phonoaudiology, University of Fortaleza, Fortaleza, Brazil; 3 Department of Physical Therapy, Faculty of Medicine, Federal University of Ceará, Fortaleza, Brazil

**Keywords:** Parkinson’s disease, Balance dysfunction, Vestibular disease, Rehabilitation, Movement disorder

## Abstract

**Introduction:**

Postural balance impairment can affect the quality of life of patients with Parkinson’s disease. Previous studies have described connections of the vestibular system with postural functions, suggesting a potential participation of the basal ganglia in receiving vestibular stimuli. This systematic review aims to summarize the evidence on the effectiveness of vestibular rehabilitation on postural balance in patients with Parkinson’s disease.

**Methods:**

A systematic review was conducted using the electronic databases: PubMed, Embase, Scopus and PEDro. The study selection was independently conducted by two reviewers, and disagreements were evaluated by a third reviewer. The included studies had no restrictions on publication dates or languages and the last update occurred in July 2023.

**Results:**

From the 485 studies found in the searches, only 3 studies were deemed eligible for the systematic review involving a total of 130 participants. The Berg Balance Scale was described as the tool for evaluation of postural balance in all studies. The meta-analysis showed statistically significant results in favor of vestibular rehabilitation (MD = 5.35; 95% CI = 2.39, 8.31; *P* < 0.001), regardless of the stage of Parkinson’s disease. Although the effect size was suggested as a useful functional gain, the analysis was done with caution, as it only included 3 randomized controlled trials. The risk of bias using the RoB-2 was considered as being of “some concern” in all studies. Furthermore, the quality of the evidence based on the Grading of Recommendations Assessment Development and Evaluation system, produced by pooling the included studies was considered very low.

**Conclusion:**

Compared to other interventions, vestibular rehabilitation has potential to assist the postural balance of patients with Parkinson’s disease. However, the very low quality of the evidence demonstrates uncertainty about the impact of this clinical practice. More robust studies are needed to confirm the benefits of this therapy in patients with Parkinson’s disease. This study was prospectively registered in PROSPERO: CRD42020210185.

**Supplementary Information:**

The online version contains supplementary material available at 10.1186/s12883-024-03649-5.

## Introduction

Patients with Parkinson’s disease (PD) usually present postural imbalance [1, 2]. The involvement of vestibular afferents in the basal ganglia and in the integration of vestibular, visual and proprioceptive information is well described in previous studies in patients wirh PD [3–5]. Alterations in the vestibular nuclei, in the lateral vestibulospinal system and in the vestibulo-ocular reflex gain may also occur [6, 7]. Furthermore, degeneration of cholinergic neurons, the pedunculopontine nucleus complex and its thalamic efferent terminals may contribute to postural imbalance [8, 9].

Studies have shown distinct peripheral and central alterations in the vestibular function of patients with PD. Vestibulo-ocular reflex impairment has been evidenced in previous studies. Findings of unilateral peripheral vestibular hypofunction were described in the caloric test in patients with PD with lateral trunk flexion [10]. Furthermore, a reduction in gains in the anterior and posterior semicircular canals in the Video Head Impulse Test (V-HIT) [11] was also significant when compared to the control group. Unilateral and bilateral absent responses were found in cervical and ocular vestibular evoked myogenic potentials, with latencies and amplitudes being significantly lower compared to the control group [11].

Changes in vestibular function in PD can trigger postural imbalance and high rates of disability and falls [12]. Thus, rehabilitation programs have been extensively studied to overcome these issues [13]. Vestibular rehabilitation is a treatment option for several cases of dizziness, instabilities and postural imbalance [14]. We questioned whether vestibular rehabilitation would be a therapy for body balance in patients with PD. Our hypothesis was that vestibular rehabilitation would be effective as a therapy for postural balance in PD, when compared to other interventions.

This type of therapy was introduced in the 1940s [15] and encompasses neuroplasticity mechanisms to reduce symptoms, repair and adapt functions [16]. Vestibular rehabilitation exercises stimulate visual stabilization, reduce sensitivity during head movements and increase vestibulo-visual interaction. The stimulation of the vestibulo-ocular reflex promotes changes in neuronal responses to head movements, enables better head alignment and contributes to the function of postural balance. Furthermore, vestibulospinal reflex exercises provide improved static and dynamic stability and functional balance in daily life situations [16].

Vestibular rehabilitation therapy needs to be planned according to the signs and symptoms of each patient, regardless of the peripheral or central findings of the vestibular evaluation [17]. However, there is little evidence about its application in patients with PD. Establishing the effectiveness of vestibular rehabilitation on postural imbalance in PD is important for a better prognosis. This systematic review aims to summarize the evidence on the effectiveness of vestibular rehabilitation on postural balance in PD patients in comparison with other interventions.

## Methods

### Review protocol and registration

This study is a systematic review aimed evaluating the effectiveness of vestibular rehabilitation on postural balance in patients with PD. This review protocol was registered in International Prospective Register of Systematic Reviews (PROSPERO) (CRD42020210185). This study was conducted according to Preferred Reporting Items for Systematic Review and Meta-analyses (PRISMA) guidelines [18].

### Eligibility criteria

The eligibility criteria were based on the following PICOS – Population (Parkinson’s disease patients), Intervention (vestibular rehabilitation), Comparison (other interventions), Outcome (postural balance) and Study type (randomized controlled trials) [18]. We included studies in which participants were diagnosed with PD according to the Movement Disorder Society Clinical Diagnostic Criteria [19]. Studies including patients with benign paroxysmal positional vertigo, dementia, or other neurological disorders (e.g., stroke, essential tremor, ataxias, or amyotrophic lateral sclerosis) were excluded. Vestibular rehabilitation was defined as a therapy to improve postural balance and was included in the intervention group, with exercises that moved the eyes, head, body and provoked sensory conflict between the vestibular, visual and somatosensory systems. The exercises included neuroplasticity mechanisms such as adaptation, habituation, and/or substitution. The use of several protocols was considered, with or without virtual or augmented reality and exergaming support. Vestibular rehabilitation associated with physical activities, strength training (weight training, functional training), aerobic training, Tai Chi, yoga, pilates, dance, aquatic exercises, multimodal and multicomponent exercises were not included.

We included any comparator groups such as no intervention, placebo, medication, balance training program, and health education guidelines. To be included, studies should present data on postural balance (scores) at the beginning and the end of vestibular rehabilitation program in the intervention and control groups. The selected studies should present at least one of the following tools for measuring balance outcomes: the Romberg test, Berg Balance Scale (BBS), Tinetti’s balance and mobility scale, static and dynamic posturography, Functional Range Test (FR), Mini Balance Evaluation Systems Test (Mini-BESTest), Time Up and Go Test (TUG), Dynamic Gait Index (DGI), vectonystagmography, subjective visual vertical, dynamic visual acuity, 2-Minute Walk Test (2MWT) and Activities-specific Balance Confidence (ABC) scale. The additional outcomes, risks of falls arising from the postural balance score at the beginning and end of vestibular rehabilitation and comparison of differences between the intervention and control groups were also extracted. The narrative synthesis of eligible studies was performed according to the characteristics of the vestibular rehabilitation intervention, sample size and outcome measure.

### Search strategy

The PubMed, Embase, Scopus and Physiotherapy Evidence Database (PEDro) electronic databases were searched. The search used the terms MeSH. The combinations of the search terms and Boolean operators were performed by an independent librarian. The following search terms were used: Parkinson, Parkinson disease, Parkinson’s disease, Parkinson’s patient, rehabilitation vestibular, central compensation, central clearing, adaptation, habituation, replacement, vestibular, vestibular function test, functional readaptation, readaptation, equilibrium, postural balance, body equilibrium, body sway, musculoskeletal equilibrium, postural equilibrium, Instability, and postural instability. The last update occurred in July 2023 and the randomized controlled trials included had no restrictions on publication dates or languages. The full search strategy is shown in Supplementary Material 1.

### Study selection

The first stage of study selection by titles and abstracts was independently performed by two expert reviewers in vestibular rehabilitation (CMSA, RPA) and disagreements were arbitrated by a third reviewer (PBN). The selection considered the eligibility criteria and duplicate studies were excluded. The full texts of the eligible studies and those in doubt were fully read with the same procedure as the first stage of data selection.

### Data extraction

Data regarding year of publication, authors, title, study design, participant characteristics, the vestibular rehabilitation intervention characteristics, types of control, control group activities, outcome values from study baseline to study endpoint and conclusion were extracted from each study by two independent reviewers (CMSA, RPA). Any disagreement between the two reviewers was resolved through discussion or arbitration by a third reviewer (PBN). E-mails and virtual messages were sent to some authors of studies to ask for information that was not included in the original studies.

### Risk of bias assessment

The Revised Cochrane risk-of-bias tool for randomized trials (RoB-2) [20] was used to assess the methodological quality of the selected trials. This analysis tool has five domains: domain 1 - risk of bias arising from the randomization process; domain 2 - risk of bias due to deviations from the intended interventions, effect of assignment to intervention or effect of adhering to intervention; domain 3 - risk of bias due to missing outcome data; domain 4 - risk of bias in measurement of the outcome and domain 5 - risk of bias in selection of the reported result. Thus, 3–7 signaling questions were asked in each domain. The response options were: yes, probably yes, probably no, no and no information. The judgment on the risk of bias for each domain was proposed by an algorithm based on signaling questions. Each domain was classified as “low risk of bias”, “high risk of bias” or “some concern”. After analysis of each domain, an overall assessment of each study was performed following the same classification criteria. The study was evaluated to have a “low risk of bias” when all domains were considered. The classification of “some concern” was determined when at least one domain was partially met or was not clearly described in the manuscript. The study was defined as “high risk of bias” if at least one domain was not met, or when it had “some concerns” in various domains. The evaluation was performed by two independent reviewers (CMSA, RPA) and disagreements were analyzed by a third reviewer (SLN).

### Quality of evidence

The quality of evidence was determined by the Grading of Recommendations, Assessment, Development and Evaluation (GRADE) system. The quality of evidence reflects the extent to which we are confident that an estimate of the effect is correct for each patient-important outcome [21]. The data evaluated encompassed five main domains: risk of bias evaluation, heterogeneity, indirectness of evidence, the imprecision of findings and potential publication bias. The evidence was classified as: “high quality” (we are very confident that the true effect lies close to that of the estimate of the effect); “moderate” (the true effect is likely to be close to the estimate of the effect, but there is a possibility that it is substantially different); “low” (confidence in the effect estimate is limited: the true effect may be substantially different from the estimate of the effect); and “very low quality” (very little confidence in the effect estimate: the true effect is likely to be substantially different from the estimate of effect). “High quality” was assigned for each included randomized controlled trial, and the evidence was then downgraded according to the analysis of each domain.

### Statistical analysis

The mean and standard deviation (SD) at baseline and follow-up were extracted for each study group. The mean difference between the scores of intervention and control groups was computed, as well as its 95% confidence intervals (CI). The random effects model was used to estimate the mean differences between the scores of intervention and control groups. Statistical heterogeneity of evaluations among studies was evaluated by Cochran’s Q test and the I^2^ inconsistency test; it was considered that values > 50% indicated high heterogeneity and p-values lower than 0.05 were considered significant. If 10 or more studies were included in the analysis of each outcome, a sub analysis was conducted considering the intervention number of sessions and length, disease stage degree according to Hoehn and Yahr classification, the patients’ medication status, and a publication bias analysis was also performed [22]. All analyses were performed using the Review Manager version 5.4 program.

## Results

**Fig. 1:**
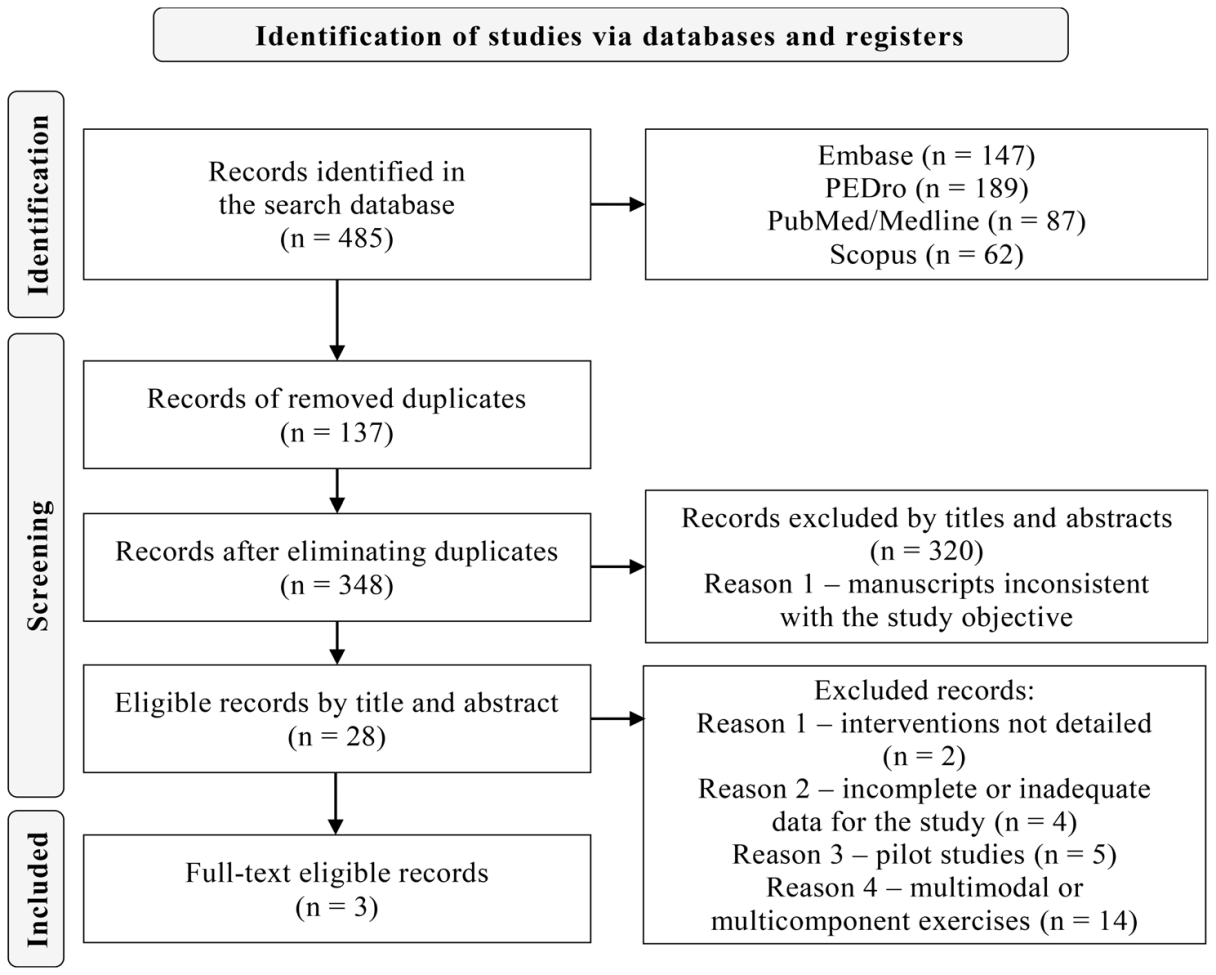
PRISMA Flowchart of the systematic review execution

Of the 485 studies obtained from the aforementioned searches, 137 were duplicates. Of the remaining 348, 320 were excluded by title and abstract and 28 were screened by reading the full text. Then, 20 were excluded according to the eligibility criteria, and 5 were analyzed by a third reviewer for discrepancies, all of which were excluded, with 3 studies remaining for the systematic review. The data were summarized and the reasons for exclusions are described in the PRISMA flowchart (Fig. 1 and Supplementary Material 2).

### Characteristics of the included studies and interventions

Of three included trials, one was conducted in Iran by Hadian et al. (2018) [23]. The other two studies took place in Italy; one by Smania et al. (2010) [24] and the other by Pazzaglia et al. (2019) [25].

The staging grade of PD was only described in two studies [23, 24], showing great variety in Hoehn and Yahr from 1 to 4. The mean duration of PD in the intervention group ranged from 3.3 to 10.3 years and from 3.7 to 8.6 years in the control group. Subjects were on PD medication in all studies [23–25] and the evaluations and interventions occurred in the “on” clinical status. Exercises in the vestibular rehabilitation intervention groups were varied, including visual exercises with eye and head movements and static and dynamic balance exercises, with eyes open and closed on stable and unstable surfaces, with and without virtual reality support [23–25]. The frequency of the interventions was three times a week and the duration ranged from three [23–25] to eight [23] weeks. The time of each session was 40 min [25], 50 min [24] and 55 to 60 min [23]. The characteristics of the included studies and interventions are described in Table 1.

**Table 1:** Characteristics of controlled clinical trials reporting vestibular rehabilitation in patients with Parkinson’s disease

Authors	Country of study	Sample Size, *N*	Mean age (SD)	H&Y	Mean years PD (SD)	Intervention	Frequency and duration of intervention	Outcomes measures (*p*-value)
Hadian et al., 2018 [23]	Iran	Intervention = 12 Control = 12	Intervention = 63.16 (8.05) Control = 63.08 (9.49)	1–4	Intervention = 3.33 (1.55) Control = 3.75 (1.60)	Vestibular rehabilitation: static and dynamic postural control exercises (eyes open and closed, stable and unstable surface) and eye movement (saccades, pursuit, vestibulo-ocular reflex) Control: warm-up exercises, muscle stretching (scapular muscles, pelvic flexors, hamstrings and gastrocnemius) exercises and standing body rotation	Frequency of three times a week for 8 weeks, with sessions of 55 to 60 min each	BBS Intervention (*p* < 0.001) Control (*p* = 0.339) FR Intervention (*p* < 0.001) Control (*p* = 0.127) 2MWT Intervention (*p* = 0.004) Control (*p* = 0.096) Total DHI Intervention (*p* < 0.001) Control (*p* = 0.674)
Pazzaglia et al., 2020[25]	Italy	Intervention = 25 Control = 26	Intervention = 72.0 (7.0) Control = 70.0 (10.0)	No given	Intervention = 7.41 (7.66) Control = 4.75 (4.41)	Vestibular rehabilitation: virtual reality exercises with movement of the eyes (fixation, saccades, pursuit), head and body (static and dynamic postural balance) Control: warm-up exercises (passive mobilization of main joints and muscular strengthening of lower limbs), motor coordination, balance training, start and stop exercises, walking training and cool-down (manipulation exercises, mobilization and respiratory)	Frequency of three times weekly for 6 weeks, with sessions of 40 min each	BBS Intervention (*p* = 0.003) Control (*p* = 0.441) DGI Intervention (*p* = 0.003) Control (*p* = 0.776)
Smania et al., 2010 [24]	Italy	Intervention = 28 Control = 27	Intervention = 67.64 (7.41) Control = 67.26 (7.18)	3–4	Intervention = 10.39 (4.76) Control = 8.63 (5.39)	Vestibular rehabilitation: balance exercises to improve feedforward and feedback postural reaction (motor actions in static and dynamic conditions, with balance exercises on different support bases) Control: exercises in active mobilization of the joints, traction, muscle stretching and motor coordination (supine, prone, sitting or standing position)	Frequency of three times a week for 7 weeks, with sessions of 50 min each	BBS Intervention (*p* < 0.001) Control (*p* = 0.062) ABC Intervention (*p* < 0.001) Control (*p* = 0.03) CFP Intervention (*p* < 0.001) Control (*p* = 0.821) Number of falls Intervention (*p* < 0.001) Control (*p* = 0.142)

*Legend* SD, mean standard deviation; H&Y, Hoehn and Yahr; PD, Parkinson’s disease; BBS, Berg Balance Scale; FR, Functional Reach; 2MWT, 2 min Walking Test; Total DHI, Dizziness Handicap Inventory-Persian Total; DGI, Dynamic Gait Index; ABC, Activities-specific Balance Confidence; CFP, self-destabilization

## Effect of interventions

### Association between BBS and vestibular rehabilitation in PD

**Fig. 2:**
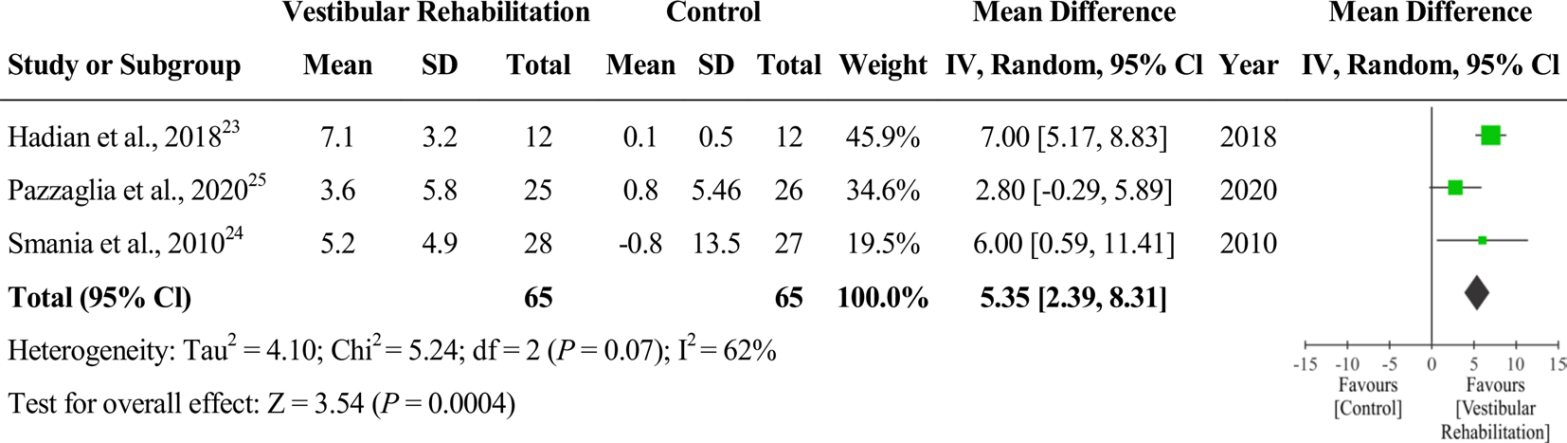
Forest plot comparing BBS between vestibular rehabilitation intervention group and control group

The BBS was described in all of the selected studies [23–25]. The pre- and post-BBS scores of the intervention group were 47.83 and 55.0 [23], 44.5 and 49.8 [24] and 45.6 and 49.2 [25], Respectively. The pre- and post-BBS scores in the control group were 45.9 and 46.0 [23], 41.8 and 41.0 [24] and 47.3 and 48.1 [25], respectively. A meta-analysis with three studies that used the BBS tool was performed comparing vestibular therapy to other interventions as comparator groups. The results are presented as a forest plot [23–25] (Fig. 2). The analysis showed a difference in favor of vestibular rehabilitation (MD = 5.35; 95% CI = 2.39, 8.31; *P* < 0.001, number of studies = 3, number of participants = 130). The heterogeneity found was not significant (*P* = 0.070) according to Cochrane’s Q test and equivalent to 62% according to the I^2^ test. Care must be taken in the interpretation of the chi-squared test, since it has low power in a meta-analysis when studies have small sample size. This level of heterogeneity is considered high [22], indicating that the effects of different interventions may differ between studies. Therefore, caution is suggested when generalizing these results.

### Other outcomes not included in the meta-analysis

The effects of other outcomes could not be analyzed because the instruments were described in only one study. However, all results in the intervention group were significant when compared before and after vestibular rehabilitation: FR [23] (MD = 7; 95% CI = 5.61, 8.38; *P* < 0.001), 2MWT [23] (MD = 23.75; 95% CI = 13.11, 34.38; *P* = 0.004), total DHI [23] (MD = -11.33; 95% CI = -15.25, -7.41; *P* < 0.001), ABC [24] (MD = 6.9; 95% CI = 4.5, 9.3; *P* < 0.001), CFP [24] (MD = 3.5; 95% CI = 1.9, 5.2; *P* < 0.001), DGI [25] (MD = 1.6; 95% CI = 0.6, 2.5; *P* = 0.003) and number of falls [24] (MD = -2.9; 95% CI = -5.3, -0.6; *P* < 0.001). Only the ABC scale showed statistically significant outcomes when comparing pre- and post-intervention in the control group: ABC [24] (MD = -1.3; 95% CI = -2.6, -0.0; *P* = 0.03).

### Risk of bias

**Fig. 3:**
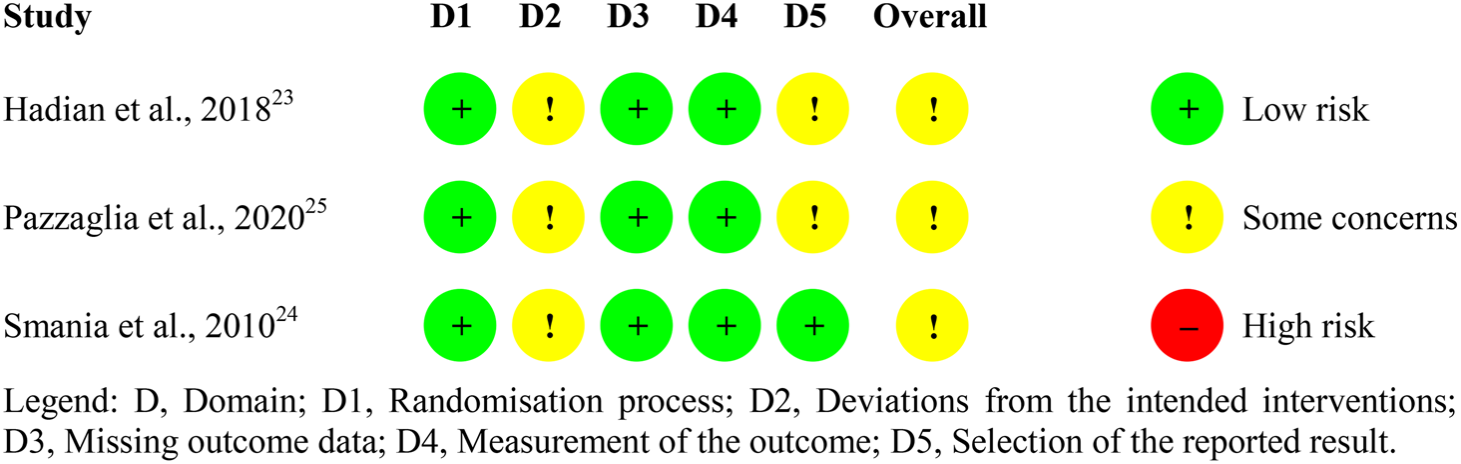
Summary of risk of bias by domain and study

Fig. 3 describes the summary of the risk of bias analysis according to the RoB2 [20]. Overall, the risk of bias was considered as being of “some concern” in all studies [23–25]. A risk of bias with “some concern” occurred in domain 2 due to the lack of information on whether a proper analysis was performed to estimate the attribution effect for the intervention [23–25]. In addition, there was no description of whether the loss of participants impacted the analysis of the results in one of the studies [24]. It was not reported whether the analysis of outcomes in domain 5 was performed according to prespecified registries [23, 25]

### Quality of evidence

The evaluation involved GRADE [21] aspects related to BBS outcomes. The quality of evidence produced by union of the included studies was considered very low (Table 2).

**Table 2:** GRADE approach: evidence profile for meta-analysis

Certainty assessment							
**Nº of studies**	**Study design**	**Risk of bias**	**Inconsistency**	**Indirectness**	**Imprecision**	**Other considerations**	**Certainty**
3	Randomized clinical trials	Serious^a^	Serious^b^	Not serious	Serious^c^	None	Very low

^a^ Risk of bias detected. ^b^ High heterogeneity not explained. ^c^ Don not have optimal information size

## Discussion

To the best of our knowledge, this is the first systematic review with meta-analysis that investigated the effectiveness of vestibular rehabilitation on postural balance in PD patients. Our meta-analysis compared vestibular rehabilitation with other exercise interventions, regardless of disease staging. Postural balance of patients with PD showed statistically significant improvements with vestibular rehabilitation [23–25].

Previous evidence in the literature also shows that patients with PD can improve postural balance with vestibular rehabilitation. The vestibular rehabilitation intervention in one of the studies was applied for 8 weeks in PD patients and compared to a control group [26]. The authors found a significant improvement in the BBS scores and in other instruments for assessing postural balance. Another study found improvement in balance in PD patients with Hoehn & Yahr scores of 3 and 4 after vestibular rehabilitation [27]. The therapy was performed in 9 sessions of 30 min each. The results sustained statistically significant improvements in all assessments and were maintained one year after the intervention. A rehabilitation program for PD patients was proposed in another study [28] for 4 weeks, in a total of 28 sessions. The results in the intervention group showed improvements in balance, gait, physical performance and trunk rotations, when compared to the control group. Other research has demonstrated beneficial results of vestibular rehabilitation in PD. One study with 24 PD patients had 12 take part in the experimental group with exercises from the Cawthorne & Cooksey protocol, which involves eye, head and body movements. Rehabilitation was carried out in 60-minute sessions, three times a week, for 12 weeks. The control group (12 patients) received the usual treatment during the research period. The BBS was used to assess postural balance. The experimental group had significantly higher scores in all BBS tests when compared to the control group, with statistically significant results [29]. Furthermore, a network meta-analysis investigated the efficacy of exergames and virtual reality in the rehabilitation of postural balance and gait in PD patients. We included 23 randomized controlled trials, of which 21 articles presented postural balance outcomes. Exergame and virtual reality were favorable strategies for vestibular rehabilitation. The proposed therapy improved postural balance in PD compared to treatment as usual and other active control interventions [30].

To explain our clinical findings, we first raised the hypothesis that the eye movements used in vestibular rehabilitation [23–25] favored the therapy results. The correlation of the extrinsic musculature of the eyes with the vestibular nuclear complex may have contributed to better postural balance function [31]. Exercises with eye movements may have improved the latency and accuracy of saccades, fixation and gain of pursuit movements. It is believed that changes in eye movements correlate with disturbances in the subcortical connections of the brainstem [32, 33]. The substantia nigra pars reticulata modulates saccade and pursuit movements [34]. One study evaluated pre- and post intervention oculomotor tests with 24 vestibular rehabilitation sessions in PD patients [35]. The authors observed better performance in the oculomotor function of fixation, saccadic, and pursuit movements, as well as in postural balance in a small cohort. These findings corroborate another previous study [36] in which correlations between oculomotor changes, postural balance and gait were suggested in PD patients. Another aspect to be considered for the purposes of our meta-analysis is the correlation of the basal ganglia with postural adjustment control [3–5]. Postural stability, static and dynamic balance exercises were proposed during the vestibular rehabilitation sessions [23–25], which may have contributed to the postural adjustment performance.

Vestibular rehabilitation is a therapy indicated for cases of postural imbalance, regardless of the degree of PD staging. It is known that postural instability manifests itself with greater intensity from stage 3 of the Hoehn and Yahr scale, which can trigger recurrent episodes of postural imbalance [37]. In addition, the PD phenotype of Postural Instability and Gait Difficulty (PIGD) presents postural instability and imbalance earlier than the Dominant Tremor phenotype [38]. Thus, subtypes of PIGD will probably need an earlier and more complex intervention. Some vestibular rehabilitation therapy protocols are used in clinical practice and research. The Cawthorne and Cooksey protocol [39, 40] which involves eye, head and body movements, was created to treat older adults who have vestibular disorders. Herdman’s protocol [41] encompasses an exercise program to increase vestibular adaptation, stabilization of static and dynamic posture and gaze and are indicated for unilateral and bilateral hypofunction. The Davis & O’Leary protocol [42] encompasses vertical and horizontal vestibulo-ocular reflex exercises and is indicated for patients with unilateral and bilateral hypofunction who present sensitivity to movement, ataxia and imbalance. Other possibilities, such as association of protocols, can be used in the therapeutic process for a broader approach to exercises. In addition, personalized vestibular rehabilitation exercises with the support of virtual reality [43] are also strategies used in therapy. There is currently a scarcity in the literature on the most suitable protocols depending on the stage of PD, the phenotype, and the structures affected at the peripheral or central level. The strategies and resources used in vestibular rehabilitation will depend on the signs and symptoms presented by PD patients. The exercises should be performed with the help of a professional for systematic guidance and with execution levels according to the difficulties of each patient [44].

The BBS measured in our meta-analysis, is a 14-item test scale which assesses performance completing balance tasks in different situations [45]. Each task is scored from (0–4), for a maximum of 56 points, which represents excellent balance and a score of 0 indicates severe equilibrium damage. The psychometric properties of the BBS show satisfactory internal consistency, intra- and interrater viability and construct validity [46]. According to the Movement Disorder Society [47, 48], the BBS is considered a valid, reliable and recommended scale to be applied to PD patients. Therefore, vestibular rehabilitation therapy can use the BBS as a viable evaluation instrument. For scales such as the BBS, it is essential to identify the Minimum Important Change (MIC) [49], which is the smallest change that an instrument can detect on the improvement or worsening of results. The European Physiotherapy Guideline for Parkinson’s disease [49] suggests using Minimal Detectable Alteration (MDC), which corresponds to the actual improvement of the effect, when MIC is not available. In a previous study [50], the MDC of 5/56 points (95% CI) was calculated for BBS in 35 patients with PD and Hoehn and Yahr from 1 to 4. The difference of 5 points of MDC, could be considered as a useful functional gain, since it is associated with the perception of improvement of the patient, which corroborates the results of our meta-analysis (DM = 5.35; 95% CI = 2.39, 8.31). In any case, our results should be interpreted with caution, as they only included three randomized clinical trials and the risk of bias was considered as being of “some concern” in all studies. Thus, a larger evidence base is needed to validate this clinical significance. In addition, according to the GRADE criteria, our systematic review provided evidence of very low quality for the use of vestibular rehabilitation in postural balance in patients with PD. Thus, the actual effects may have been distorted from the estimated effects. The classification of the GRADE instrument was considered “serious” in relation to “risk of bias”, “inconsistency” and “imprecision”. The three studies that comprise the meta-analysis of the BBS outcome presented a risk of bias classified as “some concern” bias. In relation inconsistency, it was shown that the effect and magnitude of the intervention varied between primary studies. Detection was carried out based on both the overlapping confidence intervals of the studies included in the meta-analysis and the high heterogeneity. In the criteria of imprecision, the number of patients included in the analysis was low (*n* = 130), so optimal information size was not achieved [21, 51]. The high heterogeneity of our review (I² = 62%) also requires care in generalizing the results in the PD population. Possible reasons or explanations could be considered, some of them regarding clinical features and another about methodological differences between the included studies. The patients who composed the samples of the included studies differed in terms of disease staging (Hoehn and Yahr stage), as well as time since diagnosis (Table 1). The interventions applied also showed differences in their composition. The protocols included virtual reality [25], balance exercises to improve feed forward and feedback postural reaction [24] and adaptation exercises (vestibulo-ocular reflex) [23], constituting a factor which may have influenced the differences in BBS responses.

Another important aspect to be considered in our systematic review and meta-analysis was the risk of bias. Blinding of participants and outcome assessors was reported in only one of the studies [24]. The lack of description of blinding in the other two studies [23, 25] may have interfered with the analyzed results. In addition, it was not described whether there was an intention-to-treat analysis to maintain group randomization [23, 25].

## Strengths and limitations

A strength of this study is that the study selection and data extraction process were conducted by two independent reviewers. Another point to be highlighted is that no co-intervention with vestibular rehabilitation was considered in any of the included studies [23–25]. Thus, the therapy effectiveness could be analyzed without any confounding factors. However, some limitations can be mentioned. The studies samples [23–25] were small, which may have impacted the generalizability of the results. Second, the heterogeneity of the studies [23–25] was substantial, and it was not possible to explore it with sensitivity analyses due to the low number of included studies. Finally, the intervention programs [23–25] were not uniform and the meta-analysis was performed regardless of the Hoehn and Yahr stage.

### Implications

The evidence from this study has important clinical implications. Identifying patients with postural imbalance in PD is essential for therapeutic referral. There is a need to guide health professionals about vestibular rehabilitation as a possibility for treating postural imbalance in PD. Regarding the implications of the research, further clinical trials are needed to prove the efficacy of vestibular rehabilitation in patients with PD. It is essential that studies designate which exercises are most appropriate for postural imbalance, at each stage of PD and for each phenotype. Specific strategies and protocols, personalized vestibular rehabilitation, and the use of technological resources, such as virtual reality, also need to be validated in this population. The effect size of vestibular rehabilitation on the postural balance of patients with PD in the short and long term becomes essential to evaluate the clinical outcome. Public policy reformulations may be necessary in some countries in order for PD patients to have access to this therapy.

## Conclusion

The present study demonstrated that vestibular rehabilitation has the potential to assist the postural balance of PD patients compared to other interventions. However, the very low quality of the evidence shows uncertainty about the impact of this clinical practice on the studied population. Vestibular rehabilitation interventions in PD should be applied with caution by health professionals until larger, well-conducted studies can confirm their benefits and determine their true effect size.

### Electronic supplementary material

Below is the link to the electronic supplementary material.

Supplementary Material 1

Supplementary Material 2

## Data Availability

All data generated or analyzed during this study are included in this article. Further enquiries can be directed to the corresponding author.

## Abbreviations

2MWT: 2-Minute Walk Test
ABC: Activities-specific Balance Confidence
BBS: Berg Balance Scale
CI: Confidence Intervals
DGI: Dynamic Gait Index
FR: Functional Range Test
GRADE: Grading of Recommendations Assessment, Development and Evaluation
MDC: Minimal Detectable Alteration
MIC: Minimum Important Change
Mini-BESTest: Mini Balance Evaluation Systems Test
PD: Parkinson’s Disease
PEDro: Physiotherapy Evidence Database
PICOS: Population, Intervention, Comparasion, Outcome, Study type
PRISMA: Reporting Items for Systematic Reviews and Meta-Analyses
PROSPERO: International Prospective Register of Systematic Reviews
ROB-2: Revised Cochrane risk-of-bias tool for randomized trials
SD: Standard Deviation
TUG: Time Up and Go Test
