# Supplementary material for: Effectiveness of vestibular rehabilitation on postural balance in Parkinson’s disease: a systematic review and meta-analysis of randomized controlled trials
Source: BMC Neurol. 2024 May 14;24:161. doi: 10.1186/s12883-024-03649-5 (PMC11092171; doi:10.1186/s12883-024-03649-5)
Supplement: Supplementary file 1 — Supplementary Material 1 [file 12883_2024_3649_MOESM1_ESM.docx]

**Additional file 1. Detailed search strategy**

**PubMed**

#1 Parkinson AND Rehabilitation AND vestibular.

#2 (“Parkinson Disease” OR “Parkinson’s Disease” OR Parkinson OR “Parkinson patient”) AND (“central Compensation” OR “central clearing” OR adaptation OR habituation OR replacement) AND vestibular.

**Embase**

#1 Parkinson AND Rehabilitation AND vestibular.

#2 (“Parkinson Disease” OR Parkinson OR “Parkinson patient”) AND (“central Compensation” OR “central clearing” OR adaptation OR habituation OR replacement) AND “vestibular disorder”.

**Scopus**

#1 (“Parkinson Disease” OR “Parkinson’s Disease” OR Parkinson OR “Parkinson patient”) AND (“vestibular function test” OR vestibular) AND (rehabilitation OR “functional readaptation” OR readaption).

#2 (Equilibrium OR “Postural Balance” OR “body equilibrium” OR “body sway” OR “musculoskeletal equilibrium” OR “postural equilibrium” OR Instability OR “postural instability”) AND Parkinson AND vestibular AND (rehabilitation OR “functional readaptation”).

**PEDro (Physiotherapy Evidence Database)**

Abstract & Title: Parkinson OR Parkinson’s Disease. Therapy: skill training. Method: clinical trial.
