# Supplementary material for: Effectiveness of vestibular rehabilitation on postural balance in Parkinson’s disease: a systematic review and meta-analysis of randomized controlled trials
Source: BMC Neurol. 2024 May 14;24:161. doi: 10.1186/s12883-024-03649-5 (PMC11092171; doi:10.1186/s12883-024-03649-5)
Supplement: Supplementary file 2 — Supplementary Material 2 [file 12883_2024_3649_MOESM2_ESM.docx]

**Additional file 2. Reasons for excluded studies**

| **Authors** | **Reasons** |
| --- | --- |
|  |  |
| Almeida QJ, Bhatt H, 2012 | 1 |
| El-Kholy WAH, Taha HM, Hamada SM, Sayed MAF, 2015 | 1 |
| Acarer A, Karapolat H, Celebisoy N, Ozgen G, Colakoglu Z, 2015 | 2 |
| Lee GH, 2016 | 2 |
| Tamlin B, McDonald K, Correll M, Sharpe MH, 1993 | 2 |
| Tramontano M, Bonnì S, Martino Cinnera A, Marchetti F, Caltagirone C, Koch G et al., 2016 | 2 |
| Bonnì S, Ponzo V, Tramontano M, Cinnera M, Caltagirone C, Koch G et al., 2018 | 3 |
| Claesson IM, Ståhle A, Lökk J, Grooten WJ, 2018 | 3 |
| Protas EJ., Mitchell K, Williams A, Qureshy H, Caroline K, Lai EC, 2005 | 3 |
| Ribas CG, da Silva LA, Corrêa MR, Teive HG, Valderramas S, 2017 | 3 |
| Van Den Heuvel MR, Kwakkel G, Beek PJ, Berendse HW, Daffertshofer A, Van Wegen EE, 2014 | 3 |
| Atterbury EM, Welman KE, 2017 | 4 |
| Cabrera-Martos I, Jiménez-Martín AT, López-López L, Rodríguez-Torres J, Ortiz-Rubio A, Valenza MC, 2020 | 4 |
| Conradsson D, Löfgren N, Nero H, Hagströmer M, Ståhle A, Lökk J et al., 2015 | 4 |
| Gandolfi M, Geroin C, Dimitrova E, Boldrini P, Waldner A, Bonadiman S et al., 2017 | 4 |
| Joseph C, Leavy B, Mattsson S, Falk L, Franzén E, 2018 | 4 |
| Liao YY, Yang YR, Cheng SJ, Wu YR, Fuh JL, Wang RY, 2015 | 4 |
| Sedaghati P, Daneshmandi H, Karimi N, Barati AH, 2016 | 4 |
| Shen X, Mak MK, 2012 | 4 |
| Shen X, Mak MK, 2014 | 4 |
| Shih MC, Wang RY, Cheng SJ, Yang YR, 2016 | 4 |
| Song J, Paul SS, Caetano MJD, Smith S, Dibble LE, Love R et al., 2018 | 4 |
| Szymura J, Kubica J, Wiecek M, Pera J, 2020 | 4 |
| Wallén MB, Hagströmer M, Conradsson D, Sorjonen K, Franzén E, 2018 | 4 |
| Wong-Yu IS, Mak MK, 2015 | 4 |

Legend:1, interventions not detailed; 2, incomplete or inadequate data for the study; 3, pilot studies; 4, multimodal or multicomponent exercises.
